# Exposure of *Salmonella* biofilms to antibiotic concentrations rapidly selects resistance with collateral tradeoffs

**DOI:** 10.1038/s41522-020-00178-0

**Published:** 2021-01-11

**Authors:** Eleftheria Trampari, Emma R. Holden, Gregory J. Wickham, Anuradha Ravi, Leonardo de Oliveira Martins, George M. Savva, Mark A. Webber

**Affiliations:** 1 Quadram Institute Bioscience, Norwich Research Park, Norwich, Norfolk NR4 7UQ UK; 2 Medical School, University of East Anglia, Norwich Research Park, Norwich, Norfolk NR4 7UA UK

**Keywords:** Biofilms, Antimicrobials

## Abstract

Most bacteria in nature exist in biofilms, which are inherently tolerant to antibiotics. There is currently very limited understanding of how biofilms evolve in response to sub-lethal concentrations of antimicrobials. In this study, we use a biofilm evolution model to study the effects of sub-inhibitory concentrations of three antibiotics on *Salmonella* Typhimurium biofilms. We show that biofilms rapidly evolve resistance to each antibiotic they are exposed to, demonstrating a strong selective pressure on biofilms from low antibiotic concentrations. While all antibiotics tested select for clinical resistance, there is no common mechanism. Adaptation to antimicrobials, however, has a marked cost for other clinically important phenotypes, including biofilm formation and virulence. Cefotaxime selects mutants with the greatest deficit in biofilm formation followed by azithromycin and then ciprofloxacin. Understanding the impacts of exposure of biofilms to antibiotics will help understand evolutionary trajectories and may help guide how best to use antibiotics in a biofilm context. Experimental evolution in combination with whole-genome sequencing is a powerful tool for the prediction of evolution trajectories associated with antibiotic resistance in biofilms.

## Introduction

Antimicrobial resistance (AMR) is a complex problem and a major threat to human and animal health^1^. Understanding how bacteria develop resistance to antibiotics is important to inform how antibiotics should be used to minimise the selection of AMR. Much of our understanding of the mechanisms of antibiotic action and resistance comes from experiments in which bacteria have been grown in liquid culture before being exposed to antibiotics. Yet, most bacteria in nature exist in biofilms, aggregated communities of cells encased in a matrix^2^. Biofilms represent a fundamentally different mode of life to planktonic cultures and studies have demonstrated extreme changes in gene and protein expression profiles from the same strains when grown in liquid or as a biofilm^3^. Many infections include a biofilm component that makes the infection difficult to treat; common examples include infections on prosthetic or indwelling devices. Biofilms are typically more tolerant to antibiotics, compared to the corresponding strain in liquid culture. One theory explaining the resistance to antibiotics of biofilms is that cells within a biofilm are metabolically inactive, and a high proportion are dormant ‘persister’ cells. In these dormant subpopulations, characterised by arrested macromolecular syntheses, the cellular targets that the antibiotics poison are often not essential, thus impeding bactericidal activity^4^. Despite this reduced metabolic rate, biofilms have been shown to be able to adapt rapidly to changing conditions, and rapid selection of mutants with improved biofilm fitness is observed when bacteria are introduced to a new niche^5–8^.

The selection of antibiotic-resistant mutants is a classic example of natural selection^9^, with initial studies of mechanisms of resistance exposing populations to very high concentrations of antibiotics and selecting for survivors. This identified ‘high-impact’ mutations that can confer a large phenotypic benefit and proved very useful for characterising cellular targets and primary resistance mechanisms. However, more recent work has found that repeated exposure to sub-inhibitory concentrations of antimicrobials can select for high-level resistance by different pathways^10,11^. This may better reflect real-world situations where low levels of antimicrobials are common. Importantly, this also allows epistatic interactions between multiple genes to be selected and for fitness costs arising from resistance mutations to be ameliorated by additional, compensatory mutations^12^.

The aim of this study was to use a laboratory evolution model as a tool for studying responses to sub-inhibitory concentrations of antibiotics. We employed an experimental biofilm evolution model and used *Salmonella* Typhimurium as a model biofilm-forming pathogen that we exposed to three clinically relevant antibiotics (azithromycin, cefotaxime and ciprofloxacin). Biofilms increase *Salmonella*’s chances of survival in hostile environments, providing resistance against antimicrobial agents. This is due to their unique structure and matrix composition, comprising of curli, cellulose, flagella and so on^13^. Recent evidence has suggested that controlling the level of biofilm formation by *Salmonella* impacts its virulence in vivo^14^.

In this study, we compared drug-exposed biofilm lineages to non-exposed controls and planktonic lineages. We measured changes in antibiotic resistance, biofilm capacity and pathogenicity, and subsequently investigated drug-specific mechanisms of resistance using genome sequencing. We observed rapid adaptation to antibiotic pressure, which often carried a major cost for biofilm formation. We finally explored the consequences of the acquisition of antibiotic resistance on pathogenicity while investigating the stability of resistance.

## Results

### A biofilm evolution model to study responses to antimicrobial stresses

**Fig. 1:**
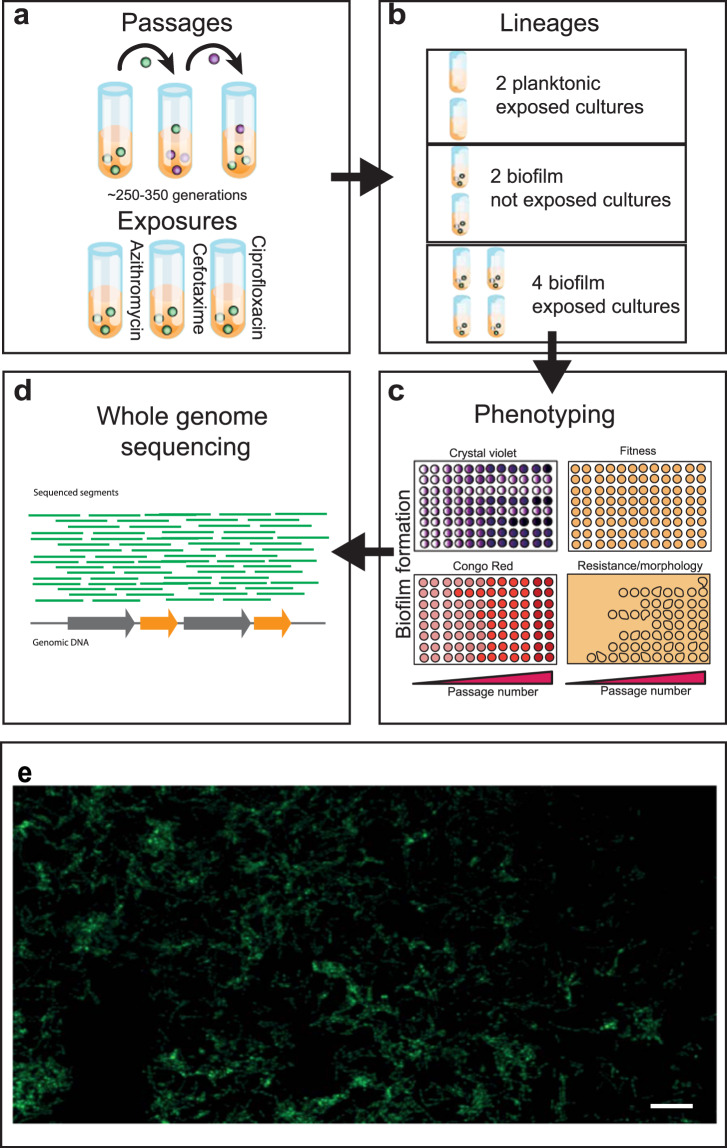
Biofilm adaptation model. **a** Sterile beads were used for the establishment of biofilms. Biofilms formed on the beads were exposed to azithromycin, cefotaxime or ciprofloxacin. **b** Eight independent lineages were included per experiment: two planktonic controls, two drug-free bead controls and four biofilm test lineages. **c** Cells were isolated every 72 h after incubation at 30 °C and phenotyped using several assays. **d** Whole-genome sequencing was used for the genetic characterisation of selected strains. **e** Confocal microscopy image (×40) of the parental strain, 14028S biofilm formed on a glass bead after 72 h. Cells were stained with SYTO9 before being visualised. Scale bar indicates 5 µM.

With relatively little known about how biofilms respond to sub-lethal exposure to antimicrobials, we studied the responses of biofilms to antibiotics and compared the results to planktonic controls using a bead model^7^. To establish *Salmonella* biofilms, we grew bacteria on glass beads in broth. The beads served as a substrate for biofilms to form on and as a biofilm transfer vehicle. With each transfer, bacteria from the biofilm community had to colonise the new beads and establish biofilms on their surface before being transferred again. This system allows longitudinal exposure of biofilms to stresses and captures all the major components of the biofilm lifecycle. After each passage, cells from biofilms were harvested and populations as well as individual representative strains were recovered and phenotypically characterised. Strains that developed resistance or exhibited altered biofilm formation were selected for whole-genome-sequencing to identify genetic mechanisms potentially responsible for these phenotypes (Fig. 1).

Biofilms were grown in the presence of three clinically important antibiotics of different classes for the treatment of Salmonellosis: azithromycin (a macrolide, targeting protein synthesis), cefotaxime (a cephalosporin targeting cell wall synthesis) and ciprofloxacin (a fluoroquinolone targeting DNA gyrase). We identified concentrations of each agent (10 μg/mL azithromycin, 0.062 μg/mL of cefotaxime and 0.015 μg/mL of ciprofloxacin) that reliably restricted planktonic growth rates to ~70% of that of unstressed control cultures. While this reduced the growth rate in exposed cultures compared to untreated controls, the incubation for 72 h allowed cultures to reach maximal growth. Analysis of numbers of cells recovered from biofilms exposed to each condition allowed us to estimate the impact of the drug exposures on the number of generations each experiment will have completed. We determined the minimum, maximum and average number of generations expected in each condition for 17 passage cycles (Supplementary Fig. 1). This showed that control biofilms completed ~305 generations (range 282–330), biofilms exposed to azithromycin completed ~289 generations (range 235–321), biofilms exposed to cefotaxime completed ~264 generations (243–280) and biofilms exposed to ciprofloxacin completed ~306 generations (265–326) in this period. Only the cefotaxime exposure resulted in a statistically significant change in the number of generations compared to control conditions. There were no significant differences in the number of cells per ml of planktonic culture after 72 h in any of the conditions. These conditions were then used for evolution experiments following an approach that has proved tractable in our previous planktonic evolution experiments^15^.

We ran three separate evolution experiments, one for each antibiotic. Each experiment included eight lineages: four drug-exposed bead lineages, two drug-exposed planktonic cultures and two drug-free bead control lineages (Fig. 1b). Evolved isolates were recovered after each passage and tested for their biofilm ability, morphology and antibiotic susceptibility. Analysis of the number of single-nucleotide polymorphisms (SNPs) present in isolates from various conditions showed no difference between treated and untreated biofilms (untreated isolates had an average of 29 SNPs compared to the parental strain, whereas isolates from treated biofilms had an average between 24 and 35 SNPs; Supplementary Fig. 2). This supports our estimation that similar numbers of generations were achieved in treated biofilms to controls.

### Model validation and response to antibiotics

**Fig. 2:**
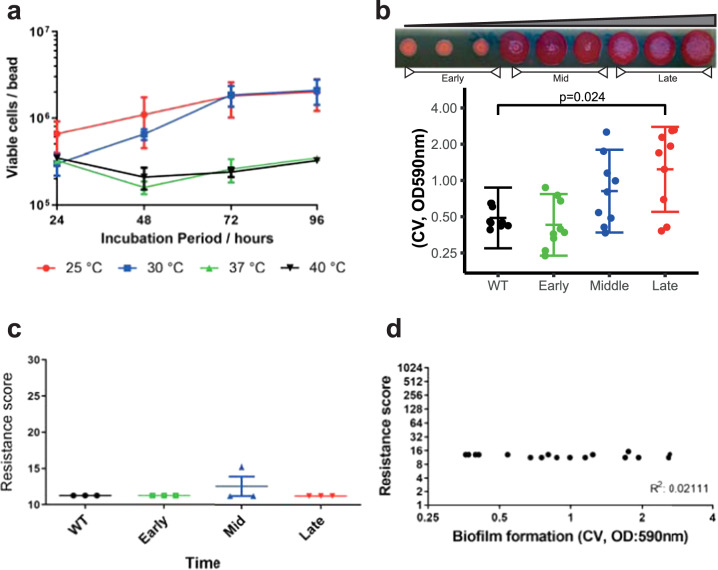
Biofilm model validation. **a** Optimal conditions were determined by the strain’s capacity to form biofilms over time, at different temperatures. Maximum cell carriage was achieved at 72 h at either 25 or 30 °C. Dots indicate average from four replicates and error bars show standard error. **b** Biofilm formation in control lineages increased over time as seen by visualisation on Congo red-agar plates and by the Crystal Violet assay (OD: 590 nm). Each dot represents single-cell strains isolated from different timepoints; experiments included three lineages each with three independent replicates. Error bars reflect estimated ± one standard error. **c** Control biofilms adapted without drug stress over time showed no changes in antimicrobial susceptibility (shown as ‘resistance score’; an additive value of all MICs determined for each strain). Data shown are from control lineages from a representative experiment. **d** No correlation was observed between biomass formation (calculated using the CV assay) and resistance score. Data shown are aggregated from the control lineages from all three drug exposure experiments.

To determine the appropriate conditions for the evolution experiments, we measured biofilm formation by *S*. Typhimurium 14028S in lysogeny broth (without salt) after 24, 48, 72 and 96 h at 25, 30, 37 and 40 °C, respectively. Biofilm formation was determined by measuring colony-forming unit (c.f.u.) per bead (Fig. 2a). Over 72 h the highest amount of biomass formed (~10^6^ c.f.u./bead) was after incubation at 25 or 30 °C; this was stable and consistent. Therefore, we ran the evolution experiments at 30 °C with a passage period of 72 h.

To confirm the model selects for the evolution of increased biofilm formation, we phenotyped isolated single cells from drug-free bead-control lineages. We observed an incremental increase in biofilm formation with colonies isolated from the drug-free control beads forming markedly larger and wrinklier colonies on Congo red (CR) plates and producing more than three times as much biomass over the course of the experiment (Fig. 2b). This confirmed that the model strongly selects for adaptation to produce biofilms with increased biomass over time in the absence of any stressor. No change in biofilm formation was seen in planktonic lineages after passage in drug-free media. Sequencing of isolated strains from biofilms adapted to produce more biofilm identified a missense substitution within CytR (Gly569Ser) in multiple independent lineages. Loss of CytR function is known to increase biofilm formation via increased CRP levels.

To investigate whether mutations that alter biofilm formation over time had an effect on susceptibility to antimicrobials, we isolated multiple single strains from populations from each of three time points across the experiment (early, mid and late) and measured the minimum inhibitory concentrations (MICs) of a panel of antimicrobials (azithromycin, cefotaxime, chloramphenicol, ciprofloxacin, kanamycin, nalidixic acid, tetracycline and triclosan). We also calculated the ‘resistance score’, which represents an additive value of all MICs determined for each strain and plotted it against time (Fig. 2c). The first observation we made was that the resistance score of unexposed biofilm lineages does not change over time despite the increase in biofilm formation. In fact, no correlation was observed between the two phenotypes (biofilm formation and resistance) in control lineages passaged in the absence of any antimicrobial exposure (Fig. 2d).

### Biofilms rapidly evolve and adapt in response to sub-inhibitory antibiotic concentrations

**Fig. 3:**
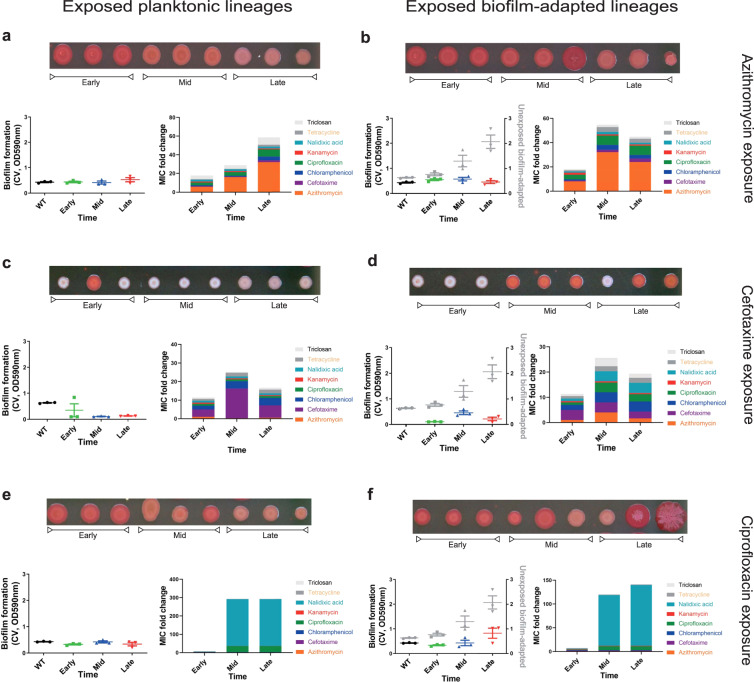
Biofilms adapt to antibiotic stress, with diverse effects on biofilm formation. Planktonic and biofilm populations, exposed to azithromycin (**a**, **b**), cefotaxime (**c**, **d**) and ciprofloxacin (**e**, **f**) were isolated at different timepoints during the evolution experiment (early, mid and late). Panels on the left show data from planktonic lineages and panels on the right from biofilm lineages. Three single isolates from each condition and timepoint were tested for their biofilm ability (measured on three separate occasions, points show the mean for the technical replicates of each repeat) and antibiotic susceptibility. For reference, biofilm formation data from isolates from control experiments with no drug exposure is overlaid on the crystal violet graphs to allow comparison (grey points on each graph). Antibiotic susceptibility of a panel of different antimicrobials was determined and visualised by stacking the average MICs (from three independent isolates per condition) for each antibiotic. All lineages, exposed to azithromycin (**a**, **b**), developed resistance to azithromycin as well as decreased susceptibility to cefotaxime, chloramphenicol, ciprofloxacin, nalidixic acid, tetracycline and triclosan. Those isolated from biofilms were, however, compromised in biofilm formation (**b**). Planktonic lineages, exposed to cefotaxime, developed resistance only to cefotaxime, whereas biofilms from the same exposure developed MDR (**c**, **d**). All lineages exposed to cefotaxime exhibited compromised biofilm formation with pale colonies on CR plates. All lineages exposed to ciprofloxacin (**e**, **f**) developed ciprofloxacin resistance and biofilm adaptation was delayed compared to the unexposed control lineages.

To test the phenotypic responses of biofilms repeatedly exposed to non-lethal concentrations of the test antibiotics, we characterised single isolates from the different timepoints for susceptibility to eight clinically relevant antimicrobials, biofilm formation and colony morphology. Phenotypic results derived from single-cell isolates from the biofilm lineages directly compared to the corresponding planktonic lineages run at the same time are presented in Fig. 3. Panels on the left show the phenotypes of strains isolated from planktonic cultures when exposed to the three antimicrobials, whereas on the right the results for the biofilm-isolated strains are presented. Biofilm formation for each strain was calculated by the Crystal Violet (CV) assay (left-hand graph in each panel) and colony morphology was also visually observed on plates supplemented with CR dye (shown above each panel). MICs were calculated using the agar dilution method for three independent colonies and the results show the fold increase in MIC for each drug (compared against the parent strain) shown as stacked bars for all the antibiotics tested in Fig. 3 (bar charts at the right-hand side of each panel).

Biofilms rapidly evolved resistance in response to all three antibiotics (Fig. 3). The time taken to select for the emergence of mutants resistant to the antibiotics was similar in most biofilm and planktonic lineages. Azithromycin selected mutants in a stepwise manner with the emergence of a population with an 8-fold MIC increase, followed by the selection of highly resistant populations with MICs of azithromycin 32 times higher than the parent strain. The resistance of the azithromycin-exposed lineages became evident at the earliest time point and was fixed by the mid-point of the experiment (Fig. 3a, b). Cefotaxime demonstrated a similar dynamic with both planktonic populations and biofilms exhibiting decreased susceptibility, which was maintained until the end of the experiment, although there was a reduction in MICs between the middle and late time points (Fig. 3c, d). Adaptation to ciprofloxacin resistance was selected by the middle of the experimental period and remained fixed up to the final timepoint in both biofilm and planktonic lineages (Fig. 3e, f). Ciprofloxacin-exposed lineages demonstrated cross-resistance to nalidixic acid (a drug in the same class), but the MICs of other drugs were impacted less than for lineages exposed to azithromycin or cefotaxime.

While the selection dynamics seemed similar between biofilm and planktonic lineages at first glance, analysis of the MICs of all the antibiotics tested revealed significant differences in the outcomes between biofilm and planktonic conditions. For instance, while planktonic populations, exposed to cefotaxime, become mainly resistant to cefotaxime, cefotaxime-exposed biofilms exhibited a broader multidrug resistance (MDR) phenotype (Fig. 3d). This pattern was consistent between independent lineages. These observations show that while the biofilms develop resistance to the selective antibiotics in a similar timeframe to planktonic cultures, some of the outcomes and underlying mechanisms are likely to be distinct.

### Development of resistance often carries a cost in biofilm formation

While it is widely accepted that increased biomass and matrix production improves the resilience of biofilms to antimicrobial stress, we observed that biofilm formation itself was heavily influenced by the selective antibiotic. For example, exposure to azithromycin selected resistance but strains under this selective pressure were severely constrained in adapting and forming better biofilms when compared to unexposed biofilms which produced much more biomass over time (Fig. 3b). Cefotaxime had a strong negative effect on biofilm formation, with biofilms exposed to cefotaxime producing less biomass than the starting wild-type strain and exhibiting a pale colony morphology on CR plates (Fig. 3d). Ciprofloxacin had less impact on biofilm formation and biofilms exposed to this drug produced increased biomass over time, although to only half the level of the control biofilms. As expected, isolates from planktonic lineages did not form better biofilms over time. In fact, cefotaxime exposure was again selected for weaker biofilms with a pale colony morphology on CR plates (Fig. 3c).

These data show that even low concentrations of antibiotics impose a strong selection for resistance to emerge in *Salmonella* biofilms, but that this carries a large cost to biofilm formation. This antibiotic pressure overrides the strong selection of the model for increased biofilm formation seen in the control lineages and the substitution within CytR seen in untreated lineages was not detected in any of the drug-exposed biofilms.

### Different antibiotics select for bespoke mechanisms of resistance

To identify the mechanisms responsible for the phenotypes described above, we carried out whole-genome sequencing on 135 populations (26) and isolated strains (109) from across the experiments and identified changes in each compared to the parent strain genome. We also used these data to infer the phylogeny of the mutants (from 63 isolates selected to represent the major phenotypes of interest and to cover different times in the exposure series for each drug) (Supplementary Fig. 3). The results showed that the different antibiotics selected for mutants followed distinct paths of adaptation, and for all antibiotics, there was a separation between biofilm (dark circles) and planktonic (light circles) lineages. With no common changes shared among the three drug exposures, it is unlikely that a universal or generic mechanism of resistance in biofilms or planktonic lineages exists.

**Fig. 4:**
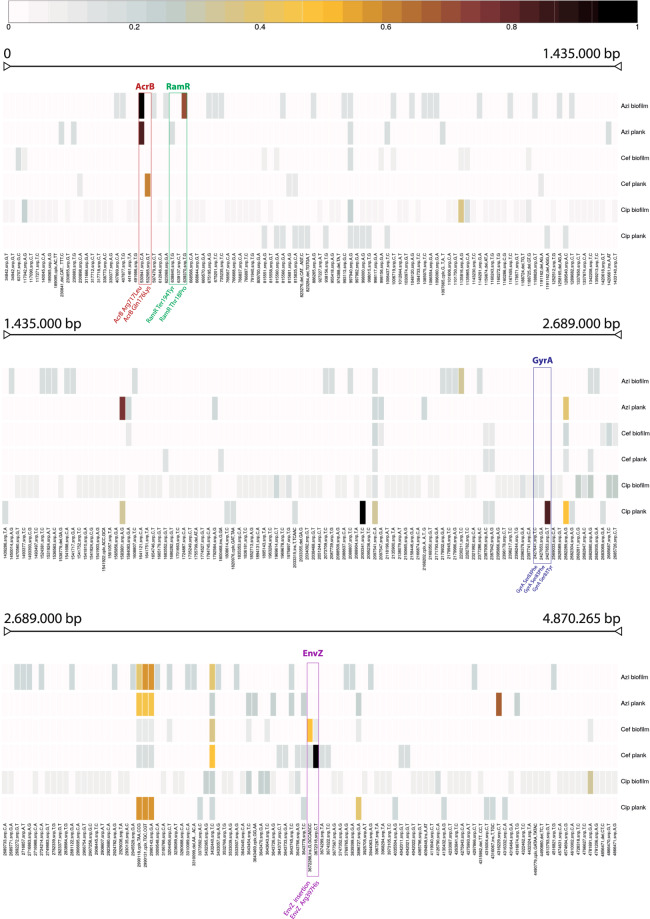
Genetic variant distribution per genomic position. For each group (a combination of antibiotic exposure and biofilm formation), we calculated the average variant presence (SNPs, insertions, deletions, complex changes, etc.). The absence of the variant in all sequenced isolates is scored as zero (white), indicating that no samples had the variant. The presence in all isolates is scored as one (black), indicating its presence in all sequenced isolates. Intermediate values represent the fraction of samples where the variant was observed. The three panels represent three continuous sections of the genome.

To identify mutations linked to the phenotypes observed, we calculated the average variant presence per environment (planktonic and biofilm) and per exposure (Fig. 4). The presence of a variant in all isolates (from independent lineages) per condition was scored as 1 (black), whereas the absence of a genomic change was scored as 0 (white). Intermediate values represent the fraction of isolates carrying the genetic change.

Among the genetic changes identified, some were identified as being responsible for drug resistance, and were often shared between biofilms and planktonic cultures. For example, under azithromycin exposure, an SNP emerged in both conditions resulting in an arginine to glutamine substitution at position 717 of the major efflux pump component AcrB. A substitution in a different position within AcrB (Gln176Lys) emerged only in planktonic cultures under cefotaxime selection. Several other substitutions within AcrB have been characterised in the past for their involvement in reduced drug susceptibility^16,17^.

Another target identified, previously linked to drug resistance was RamR, which is a transcriptional repressor of *ramA*, a global transcriptional activator that positively regulates the AcrAB-TolC pump production^18^. Mutations within RamR emerged under azithromycin exposure. Substitution in position 194 (term194Tyr) of the same protein was seen in planktonic cultures and a substitution in position 18 (Thr18Pro) emerged in the biofilm environment.

Under cefotaxime exposure, mutations within the periplasmic protein EnvZ emerged in multiple locations within the protein and were unique for this condition. EnvZ is an osmolarity-sensor protein, attached to the inner membrane of the cell. It functions both as a histidine kinase and a phosphatase, exerting its activity by altering the phosphorylation levels of its cognate transcriptional regulator OmpR. OmpR, among other functions, is responsible for differential regulation of the expression of OmpC and OmpF, two principal outer-membrane porins^19,20^, as well as curli biosynthesis through the *csgD-csgBAC* operon^21^.

Under ciprofloxacin exposure, mutants resulting in substitutions within GyrA were observed, with multiple substitutions emerging at Ser83 in both biofilm and planktonic conditions. Ser83 substitutions within GyrA are well characterised for roles in quinolone resistance^22–24^.

### Increased biomass had a limited impact on resistance

Control biofilms not exposed to drugs adapted and formed better biofilms over time (Fig. 2b). However, while this increase in biomass production was significant with adapted biofilms producing ~3× as much biomass as the parent strain, this did not result in reduced drug susceptibility (Fig. 2c, d). To further examine the relative impact of prior exposure to a specific drug versus biomass formation on survival to drugs in a biofilm, we selected a set of four strains and examined their ability to survive exposure to ciprofloxacin when grown as a biofilm. We compared the parental strain: a planktonic isolate that had been repeatedly exposed to ciprofloxacin, which demonstrated ciprofloxacin resistance but wild-type biomass production (Cip-plank-L-S1); a control biofilm-adapted isolate not exposed to any drug, which demonstrated high biomass production (~6× more than wild-type) and wild-type ciprofloxacin susceptibility (biofilm-control-L-S1); and a ciprofloxacin-exposed, biofilm-adapted isolate, which was ciprofloxacin-resistant and also demonstrated some increased biomass (~3× more than wild-type) production (Cip-biofilm-L-B-S3).

**Fig. 5:**
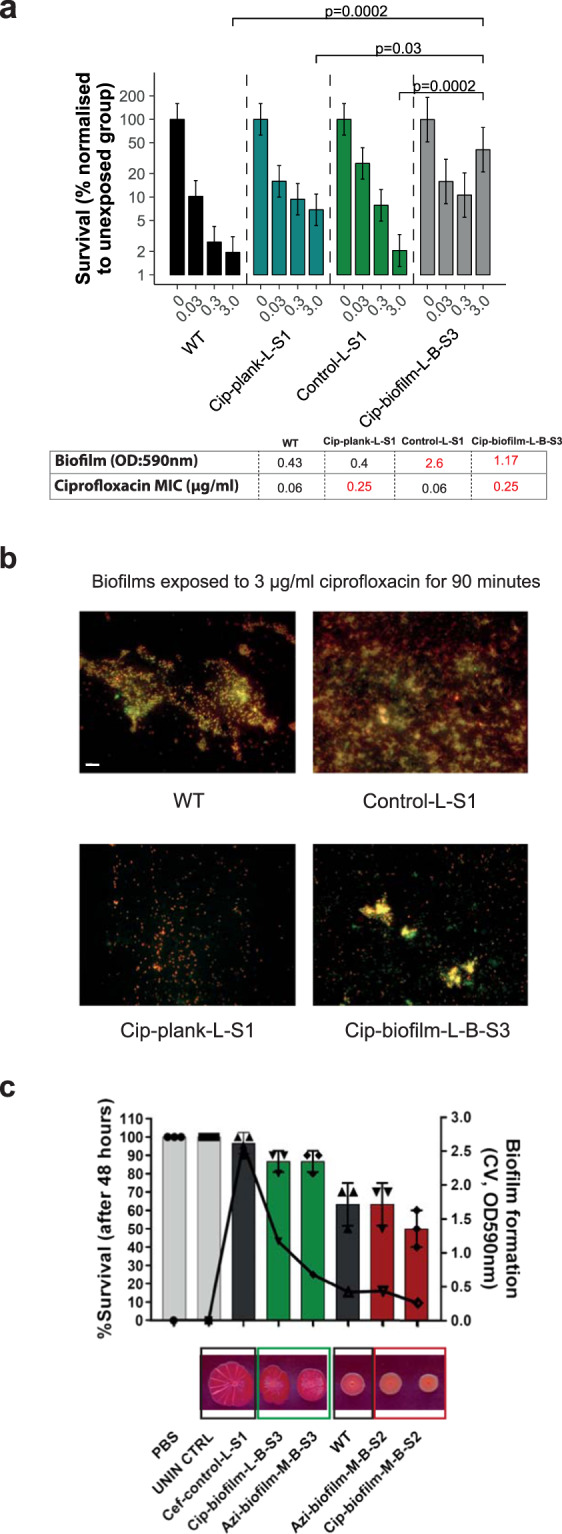
Consequences of resistance. **a** Viability within a biofilm was tested using 72-h biofilms treated with increasing concentrations of ciprofloxacin (0, 0.03, 0.3 and 3 μg/mL). The strains tested were: cip-plank-L-S1 (resistant; low-biofilm former), control-L-S1 (sensitive; good biofilm former) and cip-biofilm-L-B-S3 (resistant; good biofilm former). Only biofilms produced by cip-biofilm-L-B-S3 were significantly harder to kill with ciprofloxacin. **b** The same biofilms as in (**a**) were pre-treated with 3 μg/mL ciprofloxacin for 90 min, treated with live/dead stain and visualised. The different strains formed biofilms of variable density and an increased number of live cells was only observed in biofilms produced by the cip-biofilm-L-B-S3 strain. **c** Pathogenicity (bars) was tested in the *Galleria mellonella* infection model. Each point indicates the average number of survivors from independent experiments and the bars show the average of these. The strains tested comprised different resistance phenotypes, with diverse biofilm abilities. WT and the unexposed control-L-S1 strain were used as references. Biofilm formation (lines) was measured by the CV assay and on CR plates. Survival was directly correlated with biofilm formation, with weak biofilm formers causing more deaths in this model regardless of antibiotic resistance. Scale bar indicates 5 µM.

These isolates were allowed to form biofilms on beads before being exposed to a range of ciprofloxacin concentrations (Fig. 5a and Supplementary Fig. 4). We then determined the relative survival rate of cells within each biofilm (in relation to the corresponding untreated lineage) by counting viable cells from various adapted lineages and by live/dead staining and microscopy.

The results showed that neither being ciprofloxacin-resistant nor producing increased biomass alone allowed survival to high concentrations of the drug. Cells able to survive exposure to high levels of ciprofloxacin were only observed in the lineage that had previously been exposed to ciprofloxacin in the biofilm context even though this strain had the same MIC of ciprofloxacin as the strain recovered from planktonic exposure to ciprofloxacin (Fig. 5a). Although control biofilms made significantly more biomass than the drug-exposed biofilms, they only exhibited a mild increase in survival compared to the parental strain. Biofilms formed by the drug-exposed planktonic lineage (adapted and highly resistant to ciprofloxacin but with equivalent biofilm formation ability to the parent) also only had a mild increase in survival compared to WT biofilms. Only bacteria from the biofilm lineage exposed to ciprofloxacin exhibited a significant increase in survival. These data suggest that for optimum survival within a biofilm context neither acquisition of specific resistance to ciprofloxacin nor the ability to make improved biofilms are enough, whereas conditional exposure to the drug results in best survival when challenged. These results were confirmed by fluorescence microscopy (Fig. 5b) when biofilms of the same strains were grown on coverslips and exposed to equal amounts of ciprofloxacin.

### Development of drug resistance in biofilms impacts virulence

To test whether drug adaptation influences pathogenicity, we selected isolates from different exposures and tested their virulence using the *Galleria mellonella* infection model (Fig. 5c, bar chart). Larvae were injected with strains representing different biofilm and resistance phenotypes. Un-injected larvae as well as phosphate-buffered saline (PBS) controls were included and the wild-type strain (14028S) was used as a reference. In parallel, we measured biofilm formation and colony morphology for all cultures used to inoculate larvae using both the CV (Fig. 5c, overlaid line graph on the right axis) and the CR assay. We observed that strains with increased biofilm ability were the least infectious, whereas isolates with weaker biofilm phenotypes were more pathogenic. For example, biofilm-control-L-S1, which is a drug-free control, biofilm-adapted strain, caused the least deaths with a 95% survival rate. In comparison, cip-biofilm-M-B-S2, which is a drug-resistant but low-biofilm-forming strain, killed 50% of the larvae. Hence, adaptation to the drug exposure had no obvious advantage for pathogenicity. Our data suggest a link between pathogenicity and biofilm formation but no association to resistance.

### Resistance selected in biofilms is stable but costs to other phenotypes can be ameliorated by drug-free passage

**Fig. 6:**
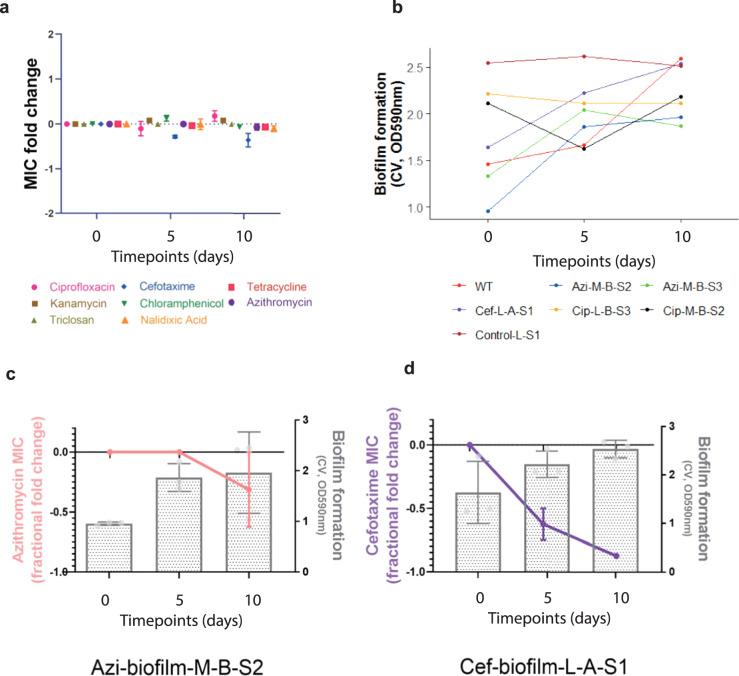
Stability of resistance. **a** Average fold change in MIC per antibiotic for seven strains was calculated after ten 24-h passages without any stressor present. For most antibiotics, resistance was stable throughout the accelerated evolution experiment, except for cefotaxime. Dots show the average fold change in MIC derived from three isolates and error bars show ±one standard error. **b** Biofilm formation increased for most of the tested strains, by the end of the experiment. Points represent modelled means in each exposure that represent the estimated proportion of cells present at each time point for each strain. **c** Individual example of an azithromycin-resistant strain, which adapted and formed better biofilm over time without losing resistance to azithromycin. Pink line shows the changes in azithromycin MIC over time (line graph, left axis). Bars (bar chart, right axis) show the average biofilm formation from three replicates. Error bars indicate standard deviation. **d** Individual example of a cefotaxime-resistant strain, which adapted to forming better biofilm but lost the resistance to cefotaxime. Purple line shows the changes in cefotaxime MIC over time (line graph, left axis). Bars and error bars are as above.

To test the stability of the phenotypes obtained during the evolution experiments, we selected seven resistant strains selected by the previous antibiotic exposures with low and high biofilm-forming abilities (see ‘Materials and methods’) and put them through a 24-hour passage, accelerated biofilm evolution experiment (Fig. 6a–d). This experiment ran for 10 days with passages every 24 hours without any antibiotics present, to test whether the resistance and biofilm patterns change over time without any selection. From each population, we isolated single strains and phenotyped them for their biofilm ability and their susceptibility against the same panel of antibiotics, as used previously. We calculated the average fold change in MIC per drug tested. In general, we observed no significant difference in drug susceptibility at the strains over time (Fig. 6a). However, their overall ability to form biofilms significantly improved (Fig. 6b). We also focused on individual strains with initially low biofilm ability and drug resistance (azi-biofilm-M-B-S2, cef-biofilm-L-A-S1) and observed that the stability of resistance was variable. In both cases, the biofilm formation of the strains improved over time. The MIC of azithromycin did not significantly change over time for the azithromycin-resistant strain (Fig. 6c, orange line), whereas the initially cefotaxime-resistant strain exhibited significantly greater susceptibility by the end of the accelerated experiment (Fig. 6d, purple line).

## Discussion

While it is known that biofilms are inherently highly tolerant to drug exposure^25–29^, little work explores how biofilms evolve in response to antimicrobials. One recent report showed that *Acinetobacter* biofilms do adapt to sub-lethal exposure to ciprofloxacin and that mechanisms of resistance were distinct to those seen in planktonic controls^30,31^. Another study showed that *Acinetobacter* and *Pseudomonas* biofilms positively respond to sub-inhibitory concentrations of the aminoglycoside tobramycin and identified parallel mechanisms of adaptation in both organisms^8^.

This study used laboratory evolution as a tool to describe the adaptation of *Salmonella* in response to three different antibiotics, identified potential pathways of resistance and explored how the environment of growth determines pathways of adaptation and potential evolutionary trajectories. We demonstrated that *Salmonella* rapidly adapts to the biofilm model and forms improved biofilms over time. Biofilms also rapidly evolved in response to sub-inhibitory exposures of antibiotics with different antibiotics selecting for drug-specific patterns of adaptation. This agrees with the recent work with *Acinetobacter* and *Pseudomonas* showing biofilms are very sensitive to low levels of antibiotics. The absence of a single, common response to the drugs tested suggests that there is not a generic antibiotic resistance mechanism in biofilms, but instead, adaptation depends on the nature of the specific stress. In line with this idea, although we showed that biofilms respond and adapt to antibiotics, this was not linked to biomass production. In fact, we identified clear trade-offs between drug resistance and biofilm formation. This pattern was not seen in the recent *Acinetobacter* and *Pseudomonas* work^8^, suggesting species and drug-specific differences in biofilm evolution pathways are important. Previous studies have associated exposure to sub-inhibitory concentrations of azithromycin and cefotaxime with a selection of mutants with inhibited biofilm formation, although only planktonic cultures were exposed in these studies^32,33^. Our data imply that although mutants adapted to these drugs have acquired a clear advantage in the presence of the antibiotic, this comes with a cost and their ability to survive in the real world may be compromised as a result.

One possible reason the biofilms exposed to drugs produced less biomass than controls could have been a lack of generations in the stressed lineages. However, we do not think this is a contributory factor for multiple reasons. First, there was only a significant reduction in the total number of generations for biofilms exposed to cefotaxime (Supplementary Fig. 1), whereas all drug exposures impaired biofilm formation. Second, even though the cefotaxime treatment resulted in fewer generations than the control, we remain confident that the smaller number of generations in this condition is not enough to explain the marked reduction in biofilm formation seen after cefotaxime exposure. For example, at the ‘late’ time point the cefotaxime lineages will have completed ~40 more generations than the control lineages have at the ‘middle’ time point; however, at these corresponding points, the cefotaxime-exposed lineages produce less biomass, whereas the controls produce significantly more biomass. Finally, the number of SNPs in different conditions was analysed; there was no difference between the conditions (Supplementary Fig. 2), so a lack of ‘mutational supply’ due to the reduced number of generations is not likely to explain the impaired biofilm formation in all drug-exposed lineages.

To study the genetic basis for the phenotypes observed, we carried out whole-genome sequencing on several strains based on their ability to form biofilms, their response to antimicrobials or a combination of the two. Some of the mutations we identified were linked to either the biofilm or the planktonic state. We also identified changes strongly associated with resistance in both environments. These mutations fall under common mechanisms of resistance affecting the balance between influx and efflux of drugs in and out of the bacterial cell, which can lead to not only susceptibility towards the stressor but also phenomena of MDR. All three of the drugs used are known efflux substrates and over-expression of efflux pumps has been identified as a mechanism of resistance for each of them^9^.

It has been shown that altering efflux function in *Salmonella* can also result in reduced biofilm formation, which may explain the trade-off between the development of resistance and impaired biofilm formation^34^. Mutations in efflux pumps and regulators were observed in both azithromycin- and cefotaxime-exposed lineages, where biofilm was most affected by the development of resistance.

Although high-level resistance to cefotaxime and azithromycin is often a result of the acquisition of specific enzymes, in this closed system, acquisition of DNA is not possible, so cells are constrained to mutation of the core genome to generate resistant mutants. For instance, azithromycin is selected for an SNP within AcrB (R717L), a major component of the MDR efflux pump AcrB-TolC, and in RamR, which is a regulatory element of the pump. These mutations appeared in independent lineages and emerged in a stepwise manner over the course of the evolution experiment. The substitution identified in AcrB was in position 717 of the protein and was shared between planktonics and biofilms, whereas the RamR Thr18Pro was restricted to biofilms. The R717L AcrB substitution was recently linked to the emergence of azithromycin-resistant *S*. Paratyphi strain in a recent outbreak in Bangladesh^35^. Other substitutions within AcrB have been identified in the past and have been predicted to change affinity for different drugs^16^.

Alteration of efflux function is a common mechanism of resistance, and overproduction of the pump is a well-characterised mechanism of resistance in clinical isolates^36^. It is likely that the mutation within RamR (Thr18Pro) is linked to the stepwise increase in MIC we observed for the strains exposed to azithromycin and substitutions within RamR including this residue have been characterised by previous studies, implicating them in drug resistance^18^.

Exposure to cefotaxime would select for mutations within EnvZ (R397H) as well as AcrB (Q176K). As shown before, alterations in AcrB’s structure can result in increased resistance to different drugs as a result of more efficient efflux^37^. EnvZ, on the other hand, has been implicated in alterations of membrane permeability, by acting through OmpR^38^. Substitutions within EnvZ were only observed under cefotaxime exposure, with the same substitution emerging in multiple lineages in parallel (R397H). This implies a strong correlation of this substitution with the emergence of resistance in these strains. A recent study has shown a link between mutations in EnvZ under selection pressure with carbapenems and alterations in membrane permeability^38^. We hypothesise, therefore, that this substitution within EnvZ may have a direct role in alterations of membrane permeability in these strains, which may lead to reduced susceptibility. Although the EnvZ mutation emerged in parallel in both the biofilm and planktonic environments, AcrB Q176K substitution was only recovered in planktonic conditions. This may suggest different routes of adaptation between planktonics and biofilms to cefotaxime.

Ciprofloxacin exposure was selected for a wider variety of mutations with much more variation in phenotypes, indicating multiple paths of evolution and resistance. The difference in the pattern of mutations selected between the drugs is likely to reflect the mechanisms of action and resistance, with multiple known chromosomal mechanisms of ciprofloxacin resistance (including target site changes, porin loss, efflux).

To explore the trade-off between biofilm formation and resistance, we selected strains representing a variety of biofilm formation and resistance phenotypes and tested them for their ability to survive exposure to a range of ciprofloxacin concentrations. We observed that only biofilms that had been exposed to ciprofloxacin were significantly harder to kill than the parent strain. This reflects their possession of both a robust community structure and drug-specific resistance mutations that make them fitter in the specific environment. Based on these results, we hypothesise that producing more biomass alone is not a sufficient solution to survive antibiotic exposure. Highly resistant biofilms are more likely to evolve from a combination of both structural and drug-specific mechanisms.

Biofilms play a crucial role in chronic infections and our observations suggested an obvious fitness advantage of adapted biofilms over unexposed biofilm populations in terms of drug resistance. To see if this impacts virulence, we investigated the pathogenicity of strains with different resistance and biofilm profiles, using the *G. mellonella* infection model. We observed that mutations that rendered the bacteria resistant to drugs had no significant impact on pathogenicity. However, the biofilm ability of the strains was negatively correlated with pathogenicity, with strains forming the least biofilm being most virulent resulting in the lowest survival rates.

Having characterised biofilm-related resistant phenotypes, we estimated their stability in the absence of drug-selective pressure using an accelerated biofilm evolution experiment. Strains that had been exposed to ciprofloxacin and azithromycin maintained their resistance profiles over extended passaging, but formed better biofilms. In contrast, cefotaxime-exposed populations lost their acquired resistance after a few passages while they became better biofilm formers. This indicates that although the stability of resistance is highly influenced by the nature of the antimicrobial stress, bacteria can quickly adapt to a more sessile, community-oriented lifestyle in the absence of a drug. Analysis of azithromycin-exposed populations that had improved their biofilm ability identified loss-of-function mutations in cyclic di-GMP phosphodiesterase, YjcC. Cyclic di-GMP is well known for its role in biofilm formation in several organisms including *Salmonella*, which harbours 12 proteins with GGDEF and 14 proteins with EAL domains^39,40^.

In conclusion, we demonstrate here that biofilms are highly sensitive to stress from low levels of antibiotics, rapidly adapt to drug pressure and that mechanisms of resistance can incur costs to other important phenotypes such as biofilm formation itself and virulence. We also demonstrate how laboratory evolution can be a powerful tool to understand the impacts of drug exposure on bacteria in different environmental niches. Future work will focus on the characterisation of the mechanisms identified in this study for their role in resistance and biofilm formation. We believe that more studies on biofilm adaptation and evolution will help inform how best to use antimicrobials and predict how biofilms will respond to different stresses in the real world.

## Methods

### Biofilm adaptation and evolution model

*Salmonella enterica* serovar Typhimurium 14028S was used as the parent strain to initiate all biofilm experiments in this study. This strain has been used as a model for *S*. Typhimurium biofilm studies by many groups including our own and has a fully sequenced and annotated reference genome (accession number: CP001363). To study the adaptation and evolution of *Salmonella* biofilms, we adapted a model described by the Cooper group^7^. Bacteria were grown on 6 mm soda lime glass beads (Sigma, Z265950-1EA) for 72 h in Lysogeny broth (LB) with no salt, which allows a mature biofilm to form (Fig. 1). They were incubated in glass universal tubes containing 5 mL of the medium in the horizontal position, secured to an orbital shaking platform (Stuart, OU-51900-21), and incubated with mild agitation (40 r.p.m.), at 30 °C. For each passage, the beads were washed in PBS and transferred into fresh media with new sterile beads. The experiment was carried out in the presence of three clinically important antibiotics: azithromycin, cefotaxime and ciprofloxacin at a final concentration of 10, 0.062 and 0.015 μg/mL, respectively. Eight independent lineages were included per exposure: four drug-exposed biofilm lineages, two drug-exposed planktonic cultures and two unexposed, bead-only control lineages (Fig. 1). In each tube, three initially sterile beads were used, one to be transferred to the next lineage, one to be stored and one from which cells were recovered for phenotyping. For storage, one bead per passage was frozen in 20% glycerol. For phenotyping, the cells were isolated from the beads by vortexing in PBS for 30 s and then grown overnight in 1 mL of LB broth, before being stored in chronological order in deep-well plates in glycerol. The experiments were completed after 17 passages for azithromycin and cefotaxime exposure and after 24 passages for the ciprofloxacin exposure. The number of biofilm generations experienced was calculated using a previously described method^7^ by the number of passages × log_2_ (the dilution factor). The number of cells per bead (dilution factor) was ~2.5 × 10^−5^ for drug-free biofilms at the start of the experiments and ranged between ~5.2 × 10^−4^ and ~2.6 × 10^−5^ for drug-exposed lineages (Supplementary Fig. 1). This represents an average number of biofilm generations after 17 passages of 305 for control lineages and between 264 and 306 for drug-exposed lineages. The analysis of control and ciprofloxacin lineages over 70 generations showed a small increase in cell numbers over the period for both conditions (Supplementary Fig. 1). Populations from an early (first passage), middle (halfway point lineage) and late (final passage) time point were chosen for detailed study, and from each, three single colonies were isolated, sub-cultured and stored in 20% glycerol. These isolates, and their parent populations, were stored in deep-well 96-well plates and used for phenotyping by replicating the bacteria onto appropriate media to test for fitness, biofilm ability, morphology and susceptibility (replication used ‘QRep 96 Pin Replicators’, X5054, Molecular Devices). Figure 1 shows an overview of the experimental setup and phenotyping procedure.

### Model optimisation

To determine the optimum culture conditions for achieving the greatest cell carriage of *S*. Typhimurium 14028S biofilms on the glass beads, biofilms were grown in 5 mL LB without salt on 6 mm glass beads at four temperatures: 25, 30, 37, and 40 °C. The cell counts on beads grown at each temperature were determined every 24 h for a duration of 96 h. Biofilms were washed in 1 mL PBS and harvested via vortexing for 30 s. The harvested cells were serially diluted in a microtiter tray containing 180 µL PBS and 5 µL was spotted onto a square LB agar plate. The number of colony-forming units was calculated and the incubation conditions yielding the greatest number of cells were determined.

### CV assay

To measure biofilm formation, selected strains were grown overnight in LB broth and then diluted into 200 μL of LB-NaCl to give an optical density (OD) of 0.01 in microtiter plates. The plates were incubated at 30 °C for 48 h, covered in gas-permeable seals before wells were emptied and vigorously rinsed with water before staining. For staining, 200 μL of 0.1% CV was added to each well and incubated for 15 min at room temperature. The crystal violet dye was then removed, and the wells were rinsed with water. The dye bound to the cells was then dissolved in 70% ethanol and the absorbance was measured at 590 nm in a plate reader (FLUOStar Omega, BMG Labtech).

### Biofilm morphology

To visually assess biofilm morphology, we replicated isolates stored in 96 deep-well plates on 1% agar LB-NaCl plates, supplemented with 40 μg/mL CR dye. The strains of interest were diluted to a final OD of 0.01 in a microtiter plate and were then printed on the CR plates using a 96-well plate pin replicator. The plates were incubated for 48 h at 30 °C before being photographed to capture colony morphology.

### Antimicrobial susceptibility testing

To determine the minimum inhibition concentrations of antimicrobials against strains of interest, we used the broth microdilution method^41^ and the agar dilution method^42^, following the EUCAST guidelines. In both cases, Mueller–Hinton broth or agar was used.

### Extraction of DNA

To extract genomic DNA for sequencing, selected strains were grown O/N in a 96-deep-well plate in LB, at a final volume of 1.5 mL. Cells were recovered by centrifugation at 3500 × *g* and were resuspended in 100 μL of lysis buffer (5 μg/mL lysozyme, 0.1 mg/mL RNAse in Tris-EDTA, pH 8) per well. The resuspended cells were then transferred in a new semi-skirted, low-bind polymerase chain reaction (PCR) plate, secured with an adhesive seal and incubated at 37 °C, 550 × *g* for 25 min. Ten microliters of a lysis additive buffer (5% sodium dodecyl sulfate, 1 mg/mL proteinase K, 1 mg/mL RNAse in Tris-EDTA, pH 8) was added to each well and the plate was sealed with PCR strip lids before being incubated at 65 °C, 550 × *g* for 25 min. The plate was briefly centrifuged and 100 μL were moved to a new PCR plate. For the DNA isolation, 50 μL of DNA-binding magnetic beads (KAPA Pure Beads, Roche Diagnostics) were added to each well and were incubated at room temperature for 5 min. The plate was then placed on a magnetic base and the supernatant was removed by pipetting. The beads were washed three times with 80% freshly prepared ethanol. After removing the last wash, the beads were left to dry for 2 min before eluting the DNA. For the DNA elution, the plate was removed from the magnetic apparatus and 50 μL of 10 mM Tris-Cl, pH 8.5, were added to each well. The beads were pulled using the magnetic apparatus and the isolated DNA was transferred to a new PCR plate. DNA concentration was determined using the Qubit ds DNA HS Assay Kit (Q32851) following the manufacturer’s instructions.

### Whole-genome sequencing

Genomic DNA was normalised to 0.5 ng/µL with 10 mM Tris-HCl. A measure of 0.9 µL of TD Tagment DNA Buffer (Illumina, Catalogue No. 15027866) was mixed with 0.09 µL TDE1, Tagment DNA Enzyme (Illumina, Catalogue No. 15027865) and 2.01 µL PCR grade water in a master mix and 3 µl added to a chilled 96-well plate. Two microlitres of normalised DNA (1 ng total) was mixed with the 3 µL of the Tagmentation mix and heated to 55 °C for 10 min in a PCR block. A PCR master mix was made up using 4 µl KAPA2G buffer, 0.4 µL dNTPs, 0.08 µL polymerase and 4.52 µL PCR grade water, contained in the KAPA2G Robust PCR Kit (Sigma, Catalogue No. KK5005) per sample and 11 µL added to each well need to be used in a 96-well plate. Two microlitres of each P7 and P5 of Nextera XT Index Kit v2 index primers (Illumina, Catalogue No. FC-131-2001 to 2004) were added to each well. Finally, the 5 µL Tagmentation mix was added and mixed. The PCR was run with 72 °C for 3 min, 95 °C for 1 min, 14 cycles of 95 °C for 10 s, 55 °C for 20 s and 72 °C for 3 min. Following the PCR reaction, the libraries were quantified using the Quant-iT dsDNA Assay Kit, High-Sensitivity Kit (Catalogue No. 10164582) and run on a FLUOstar Optima plate reader. Libraries were pooled following quantification in equal quantities. The final pool was double-SPRI size selected between 0.5 and 0.7× bead volumes using KAPA Pure Beads (Roche, Catalogue No. 07983298001). The final pool was quantified on a Qubit 3.0 instrument and run on a High Sensitivity D1000 ScreenTape (Agilent, Catalogue No. 5067-5579) using the Agilent Tapestation 4200 to calculate the final library pool molarity. The pool was run at a final concentration of 1.8 pM on an Illumina Nextseq500 instrument using a Mid Output Flowcell (NSQ® 500 Mid Output KT v2(300 CYS), Illumina, Catalogue FC-404-2003) and 15 pM on an Illumina MiSeq instrument. Illumina recommended denaturation and loading recommendations, which included a 1% PhiX spike-in (PhiX Control v3, Illumina, Catalogue FC-110-3001).

### Bioinformatics

Sequence reads from the sequencer were uploaded onto virtual machines provided by the MRC CLIMB (Cloud Infrastructure for Microbial Bioinformatics) project using BaseMount^43^. Quality filtering of the sequence reads was performed using Trimmomatic (version 3.5) with default parameters^44^. Trimmomatic’s Illuminaclip function was used to remove the Illumina adapters. The trimmed reads were then assembled into contigs using SPAdes version 3.11.1 using default parameters^45^.

To determine SNPs between the de novo assembled *Salmonella* genomes and the parent genome, Snippy version 3.1 was used using parameters recommended in https://github.com/tseemann/snippy. The tool Snippy-core from the Snippy tool box was used to determine the core SNPs. The full genome alignment output by Snippy-core was used in subsequent phylogenetic analyses, after removal of the published reference sequence (accession number CP001363). All 4,870,267 sites were included in the analysis to avoid ascertainment bias^46^. Whole-genome-based phylogenetic trees were inferred from SNPs present in 63 sequenced isolates under the model HKY + G implemented in iq-tree^47^. All trees were arbitrarily rooted at the cultivated parental sequence 14028S for visualisation purposes, and were plotted with ggtree for R^48^. Branch lengths are given in units of substitutions/site.

To identify genomic changes potentially associated with the phenotypes observed, we subtracted changes present in our parent strain (14028S) and ended up with 392 variant locations, out of the 475 initial ones. The Glmnet R package (https://www.jstatsoft.org/article/view/v033i01) was then used for a regularised regression analysis: an elastic net regularisation^49^ on a logistic regression model was applied, where the response variable was the biofilm/planktonic status, and the 392 variants were the potential predictors. This analysis was conducted independently for each antibiotic exposure. The regularisation was based on a penalty parameter *λ*, controlling the selection of features and which was found by cross-validation. The elastic net is a combination of ridge regression and lasso regularisations, with weights of 7% lasso and 93% ridge regression penalties. These proportions were arbitrarily chosen to select variants with at least one non-zero coefficient (among the three independent analyses, one per antibiotic exposure). As a result of this analysis, 301 out of the 392 SNPs were selected.

### Viability of cells within biofilms

To determine the viability of cells within a biofilm, two approaches were used. The first approach involved growing biofilms on glass beads for 72 h. They were washed in PBS to remove planktonic cells and were then challenged with different concentrations of ciprofloxacin (0, 0.03, 0.3 and 3 μg/mL) for 90 min. Beads were washed again in PBS to remove any antibiotic and transferred into 1 mL of PBS solution to an Eppendorf tube, where they were vigorously vortexed for 1 min. The cells recovered in PBS were serial diluted and spotted onto LB plates for CFU counting the next day. For the second approach, we grew biofilms on glass slides for 72 h. The slides were washed in PBS and were challenged with ciprofloxacin (3 μg/mL) for 90 min. They were washed in PBS and stained with a solution of 12 μM propidium iodide and 300 nM of SYTO 9 for 30 min. They were washed in PBS and soaked in 70% ethanol to kill the cells before they were transferred to a slide for microscopy. Fluorescence microscopy was performed in a Zeiss Axio Imager M2.

### *Galleria* infection model

To test the pathogenicity of different mutants, we used the *G. mellonella* larvae infection model. This model has previously been shown to be a simple, quick and ethical method to compare virulence between strains of *Salmonella*^24^. Wax worms were obtained from livefoods.co.uk. Similarly, sized larvae with no signs of pupation or melanisation were chosen for injection. An initial experiment was performed to calculate the infectious dose of *S*. Typhimurium 14028S in *G. mellonella*, which determined that an inoculation with ~20,000 CFU resulted in the death of approximately half of ten larvae after 72 h. Once this had been determined, overnight cultures of each strain were diluted in PBS to replicate this inoculum concentration and 10 μL of this were injected into the second hindmost left proleg of ten larvae. To check the concentration of each inoculum, 100 μL of each dilution were also plated onto LB agar and incubated overnight at 37 °C. CFUs were counted the next day and the inoculum concentration was confirmed. Controls included in this experiment included larvae injected with PBS only and un-injected larvae. All larvae were incubated at 37 °C and were checked three times a day for 3 days to record the survival rate. The experiment was repeated on three independent occasions, with ten larvae randomly allocated per strain in each experiment. Survival was calculated as the percentage of surviving larvae 48 h after injection.

### Accelerated evolution experiments

To test the phenotypic stability of strains recovered from the initial evolution experiments, we performed an accelerated evolution experiment using six strains representing a spectrum of biofilm-forming capacities and drug resistance phenotypes (WT, control-biofilm-L-S1, azi-biofilm-M-B-S2, azi-biofilm-M-B-S3, cef-biofilm-L-A-S1, cip-biofilm-M-B-S2 and cip-biofilm-L-B-S3). The strains were resuscitated from storage by a 24-h incubation at 37 °C in LB broth. After incubation, 50 µL of broth was added to 5 mL of LB broth (without salt) containing three sterile glass beads and incubated for 24 h at 30 °C, until a biofilm was formed. Each bead was then washed in 1 mL PBS to remove planktonic and loosely adherent cells. Two beads were stored in deep-well plates containing 20% glycerol for archiving and phenotyping. The third bead was transferred to another tube of LB broth (without salt) containing three sterile glass beads and passaged. This was repeated for ten passages, storing beads at each timepoint.

Upon completion of ten passages, populations were recovered from passage five, passage ten and the parental population for each mutant. From each population, single colonies were picked after streaking out each population on LB agar and incubating for 24 h at 37 °C. Three colonies from each population were then sub-cultured in LB broth. A population and three isolates from the start, middle and end of the passage series were isolated and phenotyped for each mutant. Biofilm formation was evaluated by assessing colony morphology on CR plates and by measuring biomass using CV assays as well as counting of numbers of cells from beads. The agar dilution methodology was used to assess the MICs of antibiotics. The average of the fold MIC change per antibiotic for all strains was calculated and plotted against time. The average of biofilm formation, as determined by the CV assay, was calculated for all the strains per timepoint.

### Statistical analysis

Biofilm-forming ability was compared using a regression analysis. Differences between strains over time were analysed using linear mixed models, with a random intercept of lineage where more than one lineage was included for each strain or condition.

Surviving cell counts were compared between strains using a linear mixed model with a Poisson response, with a random intercept of replicate for over-dispersion, fixed effects of exposure and the interaction between strain and exposure, and offset by the log of the average number of cells counted in the ‘unexposed’ condition for each strain. Modelled means in each exposure were then normalised by the average number of cells across all unexposed conditions for plotting, such that the values shown represent the estimated proportion of cells that would survive each exposure for each strain. All error bars reflect estimates ± one standard error.

### Reporting summary

Further information on experimental design is available in the Nature Research Reporting Summary linked to this paper.

## Supplementary information

Supplementary Information

Reporting Summary

## Data Availability

Whole-genome sequencing data that support the findings of this study have been deposited in the Sequence Read Archive with the project number PRJNA529870

## Code availability

All software used is described in the ‘Methods’ and the data and code used for analysis that support findings of this study are freely available online (https://github.com/quadram-institute-bioscience/2020.Salmonella_biofilm/blob/master/tidy_snps.ipynb).
