# Supplementary material for: Exposure of Salmonella biofilms to antibiotic concentrations rapidly selects resistance with collateral tradeoffs
Source: NPJ Biofilms Microbiomes. 2021 Jan 11;7:3. doi: 10.1038/s41522-020-00178-0 (PMC7801651; doi:10.1038/s41522-020-00178-0)
Supplement: Supplementary file 1 — Supplementary Information [file 41522_2020_178_MOESM1_ESM.pdf]

Supplementary Information

A B

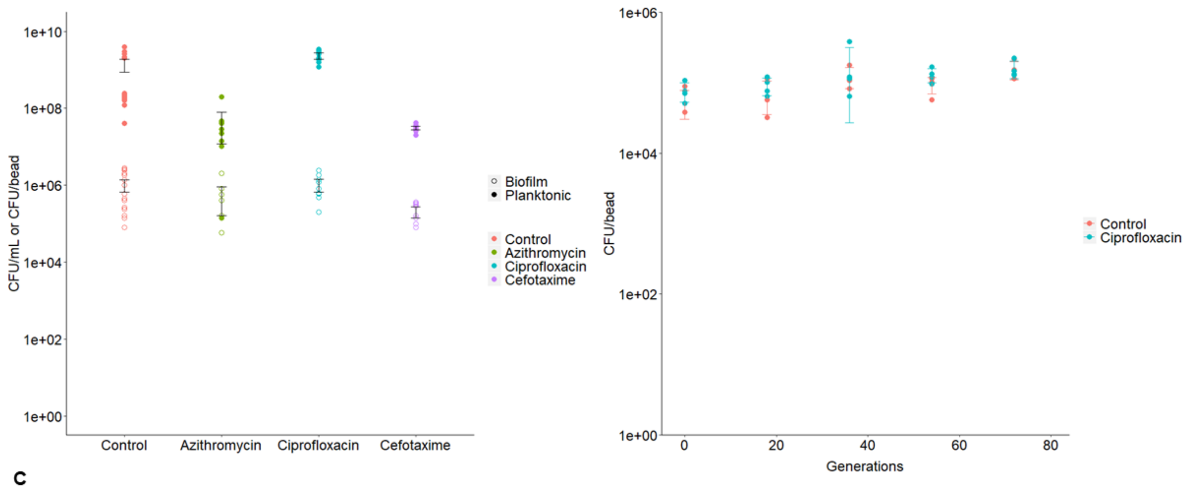

C

Number of generations (17 passages)			
	Min	Max	Average
**Control**	282	330	305
**Azi**	235	321	289
**Cef**	243	280	264
**Cip**	265	326	306

Supplementary Figure 1

Panel **A**. Numbers of viable cells in biofilms (open symbols) and in planktonic (closed symbols) recovered after exposure to the initial test conditions. Panel **B**. Number of viable cells recovered from biofilms over time in the presence and absence (control of ciprofloxacin). Panel **C**. Estimated minimum, maximum and average number of generations for biofilms incubated in all the test conditions (data for 17 passages).

A

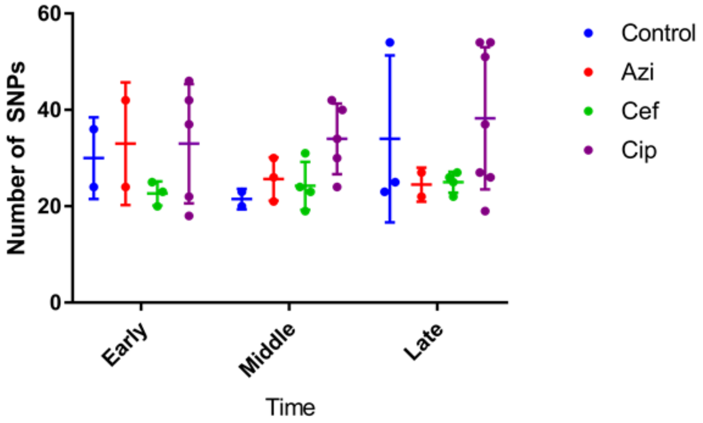

B

Average number of SNPs in isolates

	Control	Azi	Cef	Cip
Early	30	33	23	33
Middle	22	26	24	34
Late	34	25	25	38
All time points	29	28	24	35

Supplementary Figure 2

Numbers of SNPs identified (compared to the parental strain) in isolated strains from different conditions, panel **A** shows the number from each condition with the mean indicated by horizontal lines and standard deviation by error bars. Panel **B** shows a table with average numbers of SNPs in the different conditions.

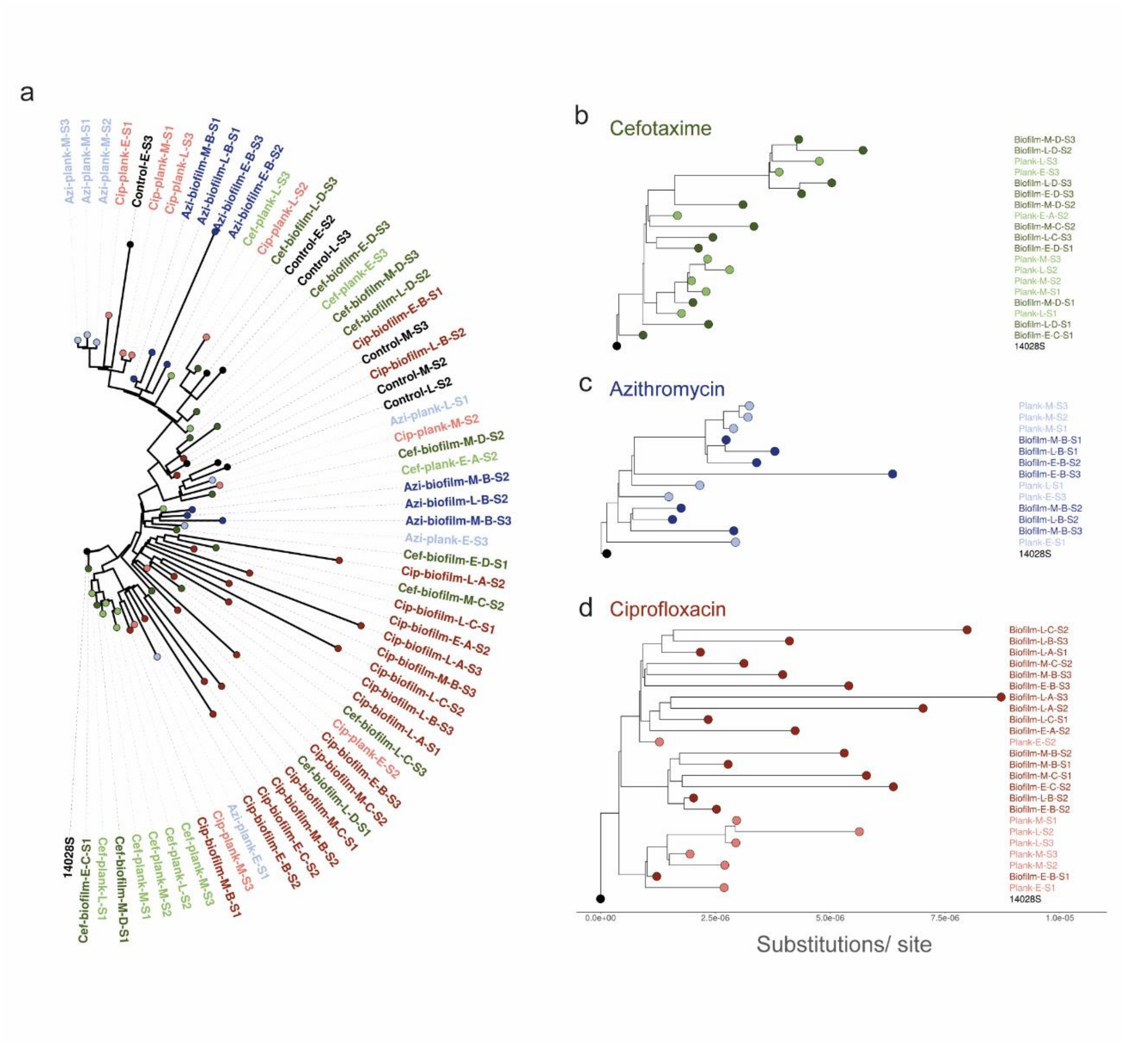

Supplementary Figure 3

Phylogenetic analysis. Panel A, Universal phylogenetic tree based on full genome alignment of all isolates, showing the divergence of strains exposed to azithromycin, cefotaxime and ciprofloxacin. Azithromycin and cefotaxime selected for mutants that followed repeatable pathways of evolution, whereas ciprofloxacin-exposed strains evolved and responded to the stress in various ways. S. Typhimurium 14028S (CP001363) was used as the reference strain and the tree was arbitrarily rooted at the cultivated parental sequence 14028S. **Panels B-D,** Individual trees were generated for cefotaxime, azithromycin and ciprofloxacin-exposed strains. Dark dots indicate biofilm lineages, light dots planktonic lineages. Phylogenetic variations between biofilms and planktonic cultures were observed, indicating unique mechanisms of adaptation between the two states.

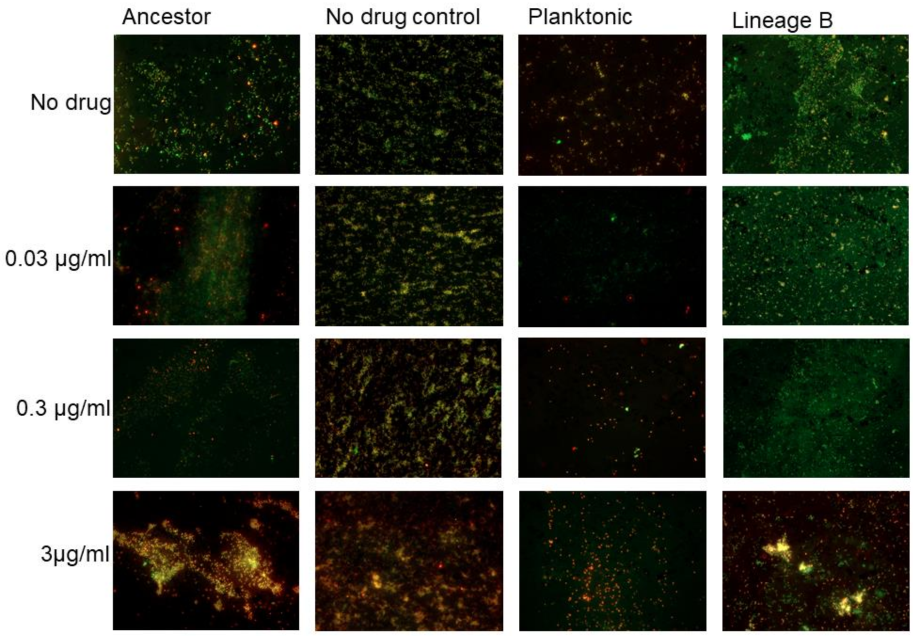

Supplementary Figure 4

Microscopy images (40X) of biofilms formed by different strains after exposure to different concentrations of ciprofloxacin, for 90 minutes. Cells were stained to differentiate ‘live’ (green) and ‘dead’ (red) cells.
